# LEISURE ACTIVITIES AND COGNITIVE IMPAIRMENT: HOW IS IT MODIFIED BY LIFE COURSE SES AND AGE AMONG CHINESE OLDER ADULTS?

**DOI:** 10.1093/geroni/igac059.055

**Published:** 2022-12-20

**Authors:** Rongjun Sun

**Affiliations:** Cleveland State University, Cleveland, Ohio, United States

## Abstract

Recent research shows that not only do life course socioeconomic status and engaging in leisure activities have independent effects on cognitive performance of older adults, but also there are significant interaction effects between them. What is less clear is whether these effects, both separate and interactions between them, vary by age among older adults. We use data from the Chinese Longitudinal Healthy Longevity Survey 2005-2014 and a Generalized Linear Mixed Model to examine these relationships. Results show that engaging in leisure activities is significantly associated with cognitive impairment even into very old ages (85+), although such associations are partially absorbed by life course SES. Furthermore, interaction effects of leisure activities and life course SES are also detected across all age groups, although they vary by specific activities. Virtually all interaction effects point to one direction: individuals of higher life course SES enjoy extra benefits from engaging in leisure activities.

